# Authors’ Response to Peer Reviews of “Predicting Escalation of Care for Childhood Pneumonia Using Machine Learning: Retrospective Analysis and Model Development”

**DOI:** 10.2196/71098

**Published:** 2025-03-04

**Authors:** Oguzhan Serin, Izzet Turkalp Akbasli, Sena Bocutcu Cetin, Busra Koseoglu, Ahmet Fatih Deveci, Muhsin Zahid Ugur, Yasemin Ozsurekci

**Affiliations:** 1 Department of Pediatrics, Hacettepe University Medical School, Gevher Nesibe Avenue, Altindag, Ankara, 06230, Turkey, 90 3051350; 2 Department of Health Information Systems, University of Health Sciences, Istanbul, Turkey; 3 Department of Pediatric Infectious Diseases, Hacettepe University Medical School, Ankara, Turkey

**Keywords:** childhood pneumonia, community-acquired pneumonia, machine learning, clinical decision support system, prognostic care decision

*This is the authors’ response to peer-review reports for “Predicting Escalation of Care for Childhood Pneumonia Using Machine Learning: Retrospective Analysis and Model Development.”*

## Round 1 Review

### Anonymous [1]

#### General Comments

*This paper [* *2* *] developed a machine learning approach that could predict community-acquired pneumonia prognosis, which is scaled into two-levels, severe or nonsevere, and identify important clinical indices, such as hypoxia, respiratory distress, age, z score of weight for age, and antibiotic usage before admission. The machine learning–based clinical decision support system tool for childhood pneumonia could provide prognostic support for case management.*

Response: Thank you for your positive summary of our work. We appreciate your recognition of the machine learning tool’s potential in supporting childhood pneumonia prognosis and case management.

#### Specific Comments

##### Major Comment

*1. To enhance the manuscript’s grounding in current research and to provide a comprehensive context for the study, the authors are recommended to incorporate an evaluation of related literature in the Introduction and Discussion sections. This could include, but not be limited to, the following studies:*

- *Liu YC, Cheng HY, Chang TH, et al. Evaluation of the need for intensive care in children with pneumonia: machine learning approach. JMIR Med Inform. Jan 27, 2022;10(1):e28934. [doi: 10.2196/28934] [Medline: 35084358]*
- *Smith JC, Spann A, McCoy AB, et al. Natural language processing and machine learning to enable clinical decision support for treatment of pediatric pneumonia. AMIA Annu Symp Proc. Jan 25, 2020;2020:1130-1139. [Medline: 33936489]*
- *Kanwal K, Khalid SG, Asif M, Zafar F, Qurashi AG. Diagnosis of community-acquired pneumonia in children using photoplethysmography and machine learning-based classifier. Biomed Signal Process Control. Jan 2024;87:105367. [doi: 10.1016/j.bspc.2023.105367]*
- *Chang TH, Liu YC, Lin SR, et al. Clinical characteristics of hospitalized children with community-acquired pneumonia and respiratory infections: Using machine learning approaches to support pathogen prediction at admission. J Microbiol Immunol Infect. Aug 2023;56(4):772-781. [doi: 10.1016/j.jmii.2023.04.011] [Medline: 37246060]*

*The readers could have a more comprehensive understanding if the authors could include a concise evaluation of the prior literature in the current manuscript.*

Response: Thank you for those invaluable articles. We have revised the Introduction and Discussion sections to include a concise evaluation of the recommended studies, along with other relevant literature, in order to enhance the readers’ understanding and to enhance alignment with the current research landscape in this niche.

*2. Considering the high stakes involved in pediatric care, particularly in intensive settings, it is critical to exam the false negative cases from the confusion matrices. Analyzing these cases for any common feature characteristics could provide insights into potential improvements in the predictive algorithm. This analysis should be clearly presented and discussed in the manuscript, emphasizing its importance in clinical decision-making.*

Response: Thank you for this important suggestion. We have carefully reviewed the false negative cases and conducted an analysis to identify any common characteristics. The analysis of false negatives of the best model “Blending-2” only revealed two false negatives, underweighting clinical features comorbidities while over-relying on the absence of hypoxia. As it only included two cases, the false negatives analysis has not been included in the Results section.

*3. The manuscript would benefit from a more detailed description of the cohort used in the study. Information on age, gender, and other clinical indices across the two groups (severe and nonsevere) would enable a better understanding of the study population. Additionally, providing the number of cases in each group would clarify the scope and scale of the study findings.*

Response: We have added a Study Population section in the Methods, providing details on the study group and the candidate variables collected. Additionally, a Study Population Characteristics section has been included in the Results, where key variables (eg, age, respiratory distress, and leukocyte count) are compared between the nonsevere and severe level of care groups (Table 2). These updates clarify the cohort’s characteristics and address your concern regarding study population details.

*4. A detailed description of the data collection process is crucial for assessing the study’s applicability in real-world clinical settings. The manuscript should explicitly state the following:*

- *How and when clinical data, including features such as hypoxia and respiratory distress, were collected (eg, at the time of admission? or within 24 hours of admission?);*
- *The time frame considered for “antibiotic usage before admission” as relevant to the prediction model: This information is essential for replicability and for future applications of the findings in clinical workflows.*

Response: We have provided a detailed description of the variables in the revised Table 1 to enhance transparency, ensuring a better understanding of how data were collected and used for the prediction model. All clinical features were encoded by pediatricians using the unstructured initial medical records at admission. For clarity and the comprehension of readers, the phrase “...candidate features from unstructured admission notes” was added to the second paragraph under the subheading of Case Definition and Patient Selection in the Methods section. Additionally, The term “recent antibiotic usage” has been clarified to indicate oral antibiotic use prescribed before admission, specifically within the 14 days preceding hospitalization. We believe these additions provide the necessary clarity and improve the replicability of the study in real-world clinical workflows.

### Reviewer E [3]

#### General Comments

*The authors have examined the medical records for 437 patients with pneumonia and created a machine learning–based classifier to determine which patients required transfer to a tertiary care center. This subject is interesting, as the predictive power of these novel statistical techniques is high and could improve the clinical care of these patients. The authors have done thorough work describing the statistical methods used in the preprocessing of the data and model development. My primary concerns in the manuscript are the lack of clinical application description, the lack of description of the time frame of the included data elements, and the lack of description regarding the patient population and outcome of interest. The following are my point-by-point comments.*

Response: Thank you for your thoughtful and detailed review of our manuscript. We appreciate your recognition of the statistical methods we used for preprocessing and model development. We acknowledge the need for improving our work in the fields that Reviewer E stated. Therefore, we have addressed each of these points as follows:

- The updated Table 1 (candidate features) provides an in-depth description of the clinical and laboratory features on how and when data collection was made (time frame), along with their clinical relevance in predicting the outcome of level of care severity. These variables were chosen based on their clinical value and ease of collection in primary care settings, allowing the model to be functional in low-resource environments.
- A new Table 2 (former Table 2 became Table 3) presents a statistical comparison between the severe and nonsevere level of care groups, focusing on the differences in demographics, clinical presentation, and laboratory values. This further highlights the factors that contribute to the outcome of interest—whether a patient requires tertiary care. The revised tables should provide a more comprehensive understanding of how the model was developed and how it applies to real-world clinical populations.
- A new subsection titled Study Population Characteristics was added under Results, where key variables were compared between groups, along with presenting the characteristics of the study population.

#### Specific Comments

##### Major Comments

###### Abstract

*The authors use the term “case management” in the Abstract and several times in the manuscript. In this context, the authors’ meaning is the decision for the escalation of care or patient transfer. However, in US-based hospital systems, case management has a different meaning, which includes largely transition to rehabilitation or nursing facilities, acquisition of home oxygen therapy, etc. I would recommend altering this term for comprehension to something like “escalation of care” or “patient triage.”*

Response: We acknowledge that the term “case management” may have different interpretations depending on the health care system. To avoid confusion, we will revise this term throughout the manuscript (including the main title) to either “prognostic care decision,” “diagnosis and treatment,” or “pneumonia management,” which are more in alignment with our study’s goal and contemporary research. Additionally, the Abstract has been substantially revised to align with the updated version of the manuscript.

*The primary outcome of interest should be included in the Abstract.*

Response: We have included a clear statement in the Abstract that the primary outcome of interest is the level of care severity, specifically focusing on the need for pediatric intensive care unit admission or advanced respiratory support.

*As detailed in the Methods section, it is crucial to describe the time frame for the included variables, to know when the algorithm could be used in clinical practice.*

Response: We specified the time frame for the data collection in the Abstract, in alignment with the changes made in texts and tables in the Methods section, ensuring that readers understand when the algorithm could be used in clinical practice. This will clarify the applicability of the model based on the retrospective nature of the data.

###### Introduction

*As the goal of the algorithm in the study is to predict which patients will need transfer to tertiary care for increasing respiratory support, more of the Introduction should focus on the management of in-hospital pediatric pneumonia, challenges, and reasons for the escalation of care.*

*I would recommend altering the sentence that describes pneumonia as easily preventable and treatable. Several of the most complicated cases in the intensive care unit are admitted with pneumonia.*

Response: Thank you for your valuable suggestions regarding the focus of the Introduction. We have revised the section to better emphasize the management of in-hospital pediatric pneumonia, including the challenges faced in recognizing and managing disease severity, as well as the reasons for escalating care. Furthermore, we have altered the sentence describing pneumonia as “easily preventable and treatable” to acknowledge the complexity of cases, particularly in intensive care settings. The revised Introduction includes the following:

1. Challenges and reasons for the escalation of care: To address this suggestion, we have expanded on the reasons for the escalation of care, providing the literature standpoint for the reasons of selecting candidate features.
2. Clarification of pneumonia’s preventability and treatability: We have revised the sentence that previously described pneumonia as “easily preventable and treatable” to better reflect the complexity of the disease.
3. More focus on the management of in-hospital pediatric pneumonia: With all respect to this comment, we kindly disagree to have more focus on in-hospital pneumonia care, as it would shift the main objective of this study, which is providing prognostic care tools for primary care settings.

###### Methods

*While great care is taken to describe the approach to data preprocessing, feature selection, and model development, I would recommend following the TRIPOD (Transparent Reporting of a Multivariable Prediction Model for individual Prognosis or Diagnosis) guidelines [* *4* *], which are validated reporting recommendations for predictive models.*

Response: Thank you for the insightful suggestion. We have reviewed the TRIPOD checklist and ensured that our manuscript adheres to these guidelines for transparent reporting of predictive models. We have uploaded the filled checklist under the section of “Upload Additional Material (for editors/reviewers’ eyes only).”

*Please provide more details regarding the hospital systems involved in this study. Are they large, academic centers or small, rural centers?*

Response: Thank you for your insightful comment. In response, we have clarified the institution in the Methods section to provide better context on the hospital system involved.

*For study inclusion, I am not familiar with the Integrated Management of Childhood Illness guidelines. Are these structured diagnostic codes captured in the electronic health record? Is it a computational phenotype?*

Response: Thank you for raising this important point. The Integrated Management of Childhood Illness guidelines are World Health Organization recommended, providing a clinical framework for diagnosing and managing pneumonia, but they are not structured diagnostic codes in the electronic health record. Physicians manually encoded clinical features from unstructured admission notes for phenotyping, rather than using a computational phenotype. This clarification has been added to the Methods section.

*Please specify what is meant by “neonatal age.”*

Response: We appreciate your suggestion for greater clarity. We have now specified that “neonatal age” refers to infants younger than 28 days of life. This has been updated in the Methods section for precision.

*Many of the variables included in the model are colinear. For example, age and weight are highly dependent on one another, and including both in the model can be detrimental. The feature selection methods may be able to discern this, but maybe not. I would recommend using only age and z score in the model.*

Response: We appreciate your insightful comments and suggestions. It appears that including both “weight” and the “weight-for-age *z* score” derived from national reference values based on age may have caused some confusion. We have clarified this issue to ensure a more coherent presentation of the candidate features. As we only included the weight-for-age *z* score (and not weight in kilogram) in our first model, no further adjustment is required in this regard. We have retained “age” as a feature because respiratory infections and disease characteristics can vary significantly across age groups. Additionally, we kept “weight-for-age *z* score” as a separate variable, as it reflects the child’s relative position among peers in the nation and serves as an indirect indicator of nutritional status.

*The time frames are not stated for the variables. For example, does “hypoxia” mean hypoxia at any time during the hospitalization? On hospital admission? In the first 12 hours? This information is vital to determine the usability of the entire model. If the model uses variables available during the entire hospitalization, the predictive ability will be high, but the usability will be low. A model that can predict right when a patient is transferred to a tertiary care center that the patient will be transferred is useless. However, a model that can predict on admission, or in the first 6‐12 hours, that a patient will require transfer is incredibly helpful. Without knowing the time frame for these variables, we cannot assess how the model could be applied in clinical practice.*

Response: We thank both reviewers for raising this important point. We agree that specifying the time frames for the variables is crucial for understanding the model’s applicability in clinical settings. In response, we have clarified the data collection process in the revised manuscript. All clinical features, including hypoxia and respiratory distress, are now detailed in the updated Table 1 and additional text in the Methods section under Case Definition and Patient Selection, with more emphasis on the relevant time frames of the features.

*Please provide clarity regarding the study outcomes. The primary outcome is described as whether the patient was referred to a tertiary care center or not. The next sentence describes “poor prognosis” as pediatric intensive care unit admission or oxygen/ventilation support. How is this outcome used? Is this a secondary outcome? Is this describing the reason for transfer? Please clarify.*

Response: Thank you for highlighting this point. We acknowledge the need to clarify the study outcomes. The primary outcome is whether the patient requires transfer to a tertiary care unit. The term “poor prognosis” refers to the reason for transfer, specifically whether the patient required pediatric intensive care unit admission or oxygen/ventilation support. This is not a separate secondary outcome, but rather the criteria used to define the primary outcome of requiring tertiary care. We have revised the manuscript to clarify that the primary outcome is the “Level of Care Severity,” along with text in the Methods section to make this distinction clear.

*As stated in the TRIPOD guidelines, you should present the amount of missingness in your data. It appears you used imputation methods for missing data. It is helpful to describe the amount of missing data that was imputed and the method for imputation.*

Response: Thank you for your valuable comment. In accordance with the TRIPOD guidelines, we agree that reporting the amount of missing data is important for transparency. We should have mentioned our imputation method while providing details about relevant features in the first submission. We have now included a detailed description of the missing data in our revised manuscript, specifying both the percentage of missing values for each variable and the total amount of missing data. To handle missing data, we used the light gradient boosting machine algorithm as an imputation method, treating missing values as a dependent variable and predicting them based on other features to avoid bias. Individual feature weights were applied accordingly. The following features had missing values: C-reactive protein (n=34, 8.2%), albumin (n=10, 2.4%), sodium (n=8, 1.9%), aspartate aminotransferase (n=16, 3.9%), and alanine aminotransferase (n=16, 3.9%). This information has been added to the revised manuscript for clarity.

###### Results

*There is a glaring lack of information regarding your study population. Please provide a table describing patient characteristics including demographics and the variables you used in the algorithm. Also, please provide a comparison between the patients who were transferred to a tertiary care center and those who were not.*

Response: Thank you for your observation. In response, we have added a detailed description of the study population in the revised manuscript. Specifically, we have included a new subsection titled Study Population Characteristics, along with a new Table 2, which presents a comparison of the demographic and clinical characteristics between the severe and nonsevere level of care groups. We have also used appropriate statistical tests to compare the characteristics of patients requiring transfer to a tertiary care unit (severe care group) versus those who did not (nonsevere group). These additions enhance the clarity of our population description and provide a comprehensive comparison of the key variables used in our algorithm.

*In imbalanced datasets, it can be more useful to measure model performance using the area under the precision-recall curve rather than the standard area under the receiver operator characteristic curve. I would recommend adding this metric.*

Response: Thank you for your insightful suggestion. We agree that in the case of imbalanced datasets, the area under the precision-recall curve (PRC) can provide a more informative measure of model performance than the standard area under the receiver operating characteristic curve. In response, we have now added the PRC of all models in the performance table. We also included a PRC plot for the blending model labeled as “Blending-2,” which incorporates the top-5 highest-ranked clinical features using the optimized CatBoost, light gradient boosting machine, and extreme gradient boosting models. The new PRC plot, along with the text explaining it in the Results section, have been added to the supplementary materials to provide a more comprehensive evaluation of the model’s performance on imbalanced data.

###### Discussion

*The Discussion, overall, focuses much more on the technical details of the data curation and model development than it does on the clinical application of the model. Much of the technical details presented are also clearly explained in the Methods section and then repeated in the Discussion. I would recommend substantial revision to the Discussion section to remove redundant information that is already contained in the Methods section, as well as the addition of how this model could be applied in a clinical setting to improve the care of patients with pneumonia.*

Response: We thank the reviewer for this valuable feedback. In response, we have thoroughly revised the Discussion section to reduce redundancy and place a greater focus on the clinical applications of the model, along with contemporary study inclusion. Specifically, we removed technical details that were previously repeated from the Methods section, such as the handling of imbalanced data with Synthetic Minority Oversampling Technique–Tomek, feature selection using Shapley additive explanations and recursive feature elimination with cross-validation, and detailed performance metrics for each algorithm.

In place of these technical details, we have expanded the Discussion to focus more on how the model can be used in a clinical setting to improve pneumonia care. We now highlight how the model can assist primary care physicians, especially those working in resource-limited environments, in identifying high-risk pneumonia cases that may require referral to tertiary care. We also put emphasis on predictive features (such as hypoxia, respiratory distress, age, weight *z* score, and complaint period) that are easy to assess in primary care, making the model highly practical for use in real-world clinical settings. Furthermore, we discuss the potential for the model to improve patient outcomes by facilitating timely care decisions, particularly in settings where advanced diagnostic tools may not be available.

*The Discussion contains no information regarding the limitations of the study. Please describe in detail the prominent limitations of the study. These should include the use of retrospective data, including only two centers, imbalanced data, challenges with clinical implementation of the model, etc.*

Response: Thank you for highlighting the need to discuss the limitations of the study in more detail. In response, we have expanded the Discussion section to include a more comprehensive account of the study’s limitations. Specifically, we now address the reliance on data from a single tertiary hospital, the potential selection bias toward severe cases, the limited sample size, and the retrospective nature of the data.

*The Discussion, and other areas of the manuscript, mention disease prevention several times. The goal of this study has nothing to do with the prevention of pneumonia, only the treatment of pneumonia and the prevention of associated morbidity and mortality. Please revise.*

Response: Thank you for pointing out the unnecessary mentions of disease prevention in the manuscript. We agree that the primary focus of the study is on the treatment of pneumonia and the prevention of associated morbidity and mortality, not the prevention of the disease itself. We have revised the entire manuscript to eliminate any mention of disease prevention where it is not relevant and have ensured that the discussion stays focused on treatment and prognosis.

###### Conclusion

*As it stands, the Conclusion is fairly long and does not focus only on the primary findings of the study. I would recommend trimming it to 2‐3 sentences that focus only on the primary findings of the study, such as the feasibility of developing this type of predictive model and the potential applications of the model to clinical practice.*

Response: Thank you for your feedback regarding the length and focus of the Conclusion. We agree that the Conclusion could be more concise and focused on the primary findings. Based on your suggestion, we have significantly shortened the Conclusion to focus solely on the primary findings of the study, namely, the feasibility of developing a predictive model for childhood pneumonia prognosis and its potential clinical applications. The revised Conclusion now highlights the key outcomes concisely.

### Minor Comments

#### Methods

*The authors describe that ensemble methods “significantly enhance the accuracy of classifications.” Please provide a reference for this statement.*

Response: We agree that providing a reference would strengthen this statement. We have now included a reference supporting our statement. Specifically, “Mahajan P, Uddin S, Hajati F, Moni MA. Ensemble learning for disease prediction: a review. Healthcare (Basel). Jun 20, 2023;11(12):1808. [doi: 10.3390/healthcare11121808] [Medline: 37372925]”

#### Results

*Please provide numbers for those who met your primary outcome of interest (transfer to a tertiary care center).*

Response: Thank you for your suggestion to provide specific numbers related to the primary outcome of interest. We have now revised the Results section to include study population characteristics along with a comparison between the severe (transferred to a tertiary care unit) and nonsevere level of care groups. The revised Results section also holds emphasis on the primary outcome of interest as follows “...Of the 437 patients analyzed, 304 patients (69.6%) met the primary outcome of being transferred required escalation of care.”

*Please provide a description of the time frame for patient transfer, for those who were transferred.*

Response: In alignment with previous comments on the inclusion of time frames to relevant data elements, we have provided a detailed description in the updated Table 1 for candidate variables. However, our dataset does not include the timing of transfers to tertiary care units. This is recognized as a limitation of the study, and the Limitation section has been extended in this regard.

#### Discussion

*It would be interesting to hear more regarding the use of this model in resource-limited settings and the benefits it could provide.*

Response: Thank you for your valuable comments, which have already enhanced our work beyond our initial vision. We share your excitement about the future potential of this work and its possible applications.

## Round 2 Review

### Anonymous

*I thank the authors for revising the manuscript.*

### Reviewer E

#### General Comments

*The authors have conducted a single-center, retrospective study evaluating the derivation and performance of a machine learning model to predict the need for transfer to a higher level of care for childhood pneumonia. The authors were provided with a substantial amount of feedback on the original submission, and although the authors’ response is detailed and comments on how all concerns were adequately addressed, the resulting manuscript is lacking in many if not most of the requested changes. The revised manuscript remains confusing to the reader and bereft of some essential elements of standard study reporting, including a basic description of the patient population and details regarding the timing of variable collection and use in the model. Due to this lack of response to the initial reviewer feedback, I am recommending rejection of this manuscript. The following are my point-by-point critiques, many of which are similar to those in my original review.*

Response: We believe that these comments may stem from a review of the earlier version of our manuscript rather than the revised submission. Each specific comment raised by the reviewer was addressed in the revised manuscript, where we carefully incorporated the requested changes and clarifications. We kindly request a review of the latest version in the JMIRx system, as it reflects these substantial updates in response to the initial feedback. As the reviewer provided some additional recommendations, we made the required changes to those in our most recent manuscript. We believe there may have been a misunderstanding or an oversight, leading to the reviewer evaluating an earlier version of our manuscript. We genuinely appreciate the time and effort the reviewer has invested in helping us improve our manuscript.

#### Specific Comments

##### Abstract

*First sentence: Please revise it to “Pneumonia is the leading cause of preventable mortality for children under five years of age.”*

Response: We have revised the first sentence of the Background section of the Abstract.

*Background: The terms “case management” and “disease prevention” are still used in the Abstract. In my initial review, I recommended revising these terms to improve study clarity, and although the authors stated in their response that they replaced these terms, they remain in the Abstract. As it stands, it is not immediately clear to the reader that the purpose of the study was to provide a tool to assist bedside clinicians to determine which patients are likely to require transfer of care to a higher-level facility for pediatric pneumonia.*

Response: Thank you for highlighting the importance of precise terminology in conveying the study’s purpose. We have already revised the entire document to address the reviewer’s initial comment/concern. We have now double-checked the revised manuscript and there is no mention of “case management” in the revised manuscript, as well as “disease prevention,” that could be misunderstood by readers.

*Methods: As it stands, it is confusing to the readers what was actually done in the study. It should be very apparent that the authors used a specific list of variables (please provide each in the Abstract) to predict the need for transfer to a larger institution using a specific type of machine learning model (ensemble). In the current version, this is difficult to discern.*

Response: We thank your attention to the need for clarity in the Abstract. We have already addressed this concern by stating “Pediatricians encoded key clinical features from unstructured medical records based on IMCI guidelines.” This line conveys that essential variables were derived from standardized guidelines without detailing each variable. Listing all variables in the Abstract would reduce clarity when considering the Abstract word limitations of this journal, especially since these variables are fully detailed in the Methods and Results sections. We believe this approach aligns with best practices for Abstract conciseness and provides sufficient information for the reader.

*Results: I would be completely clear regarding the outcome your model is predicting. After reading the paper, it is understood that “pneumonia prognosis” and “severity” actually mean required transfer to a higher level of care, but it is unclear in the Abstract. I would explicitly state “predicted transfer to a higher level of care with 77%‐88% accuracy.”*

Response: Thank you for this valuable suggestion to improve clarity. In response, we have revised the Results section of the Abstract to explicitly state that the model predicts the need for transfer to a higher level of care, specifying the accuracy range as suggested. The revised phrasing is now “The optimized models predicted the need for transfer to a higher level of care with an accuracy of 77%‐88%...” This adjustment enhances clarity and directly conveys the model’s intended outcome for readers.

##### Introduction

*Second paragraph, fifth sentence: I would recommend revising it to “However, this preventable health problem continues to be a substantial cause of mortality, especially in underdeveloped countries and regions, due to the lack of equipment and trained human resources.” There is no way to quantify it as “the most important cause of mortality.”*

Response: There is no mention of “the most important cause of mortality” in the revised manuscript. However, we noticed that it was in the first submission. We are deeply concerned that the reviewer’s second round of comments did not provide feedback on the revised manuscript.

*The term “case management” continues to be used in the Introduction, which decreases clarity for the reader.*

Response: Again, these concerns have already been addressed in the revised manuscript. There is no mention of “case management.” We kindly request the reviewer to read the revised version rather than the first submission that has been substantially changed after the reviewer’s initial comments.

*As recommended previously, I would be very specific in the Introduction that you are trying to create a tool to help bedside clinicians (typically non–intensive care physicians) decide when to transfer a patient with pneumonia to a higher level of care to prevent morbidity and mortality. As it stands, this is unclear.*

Response: Thank you for this recommendation. This point was already addressed in the revised manuscript, where we clarified the study’s goal in the Introduction. Please also refer to the Introduction section in the last paragraph, stating “We aimed to develop machine learning-based clinical decision support system tool for childhood pneumonia that can be used by physicians, particularly working in LMICs.” However, we believe including the adjective “non-intensive care” to define these physicians in detail would improve the manuscript.

##### Methods

*In my initial review, I asked the authors to clarify what is meant by neonatal age. In their response, they said they had revised the Methods to state specifically 28 days or fewer. However, in the first paragraph of the Methods, it continues to state “neonatal age.” Please revise.*

Response: Thank you for raising this point again. We did agree on this issue and corrected it in the revised manuscript as follows: “Patients younger than 28 days of age (neonatal age), older than 18 years, and those who had been hospitalized within the last 14 days were excluded.” Preserving the neonatal age in this sentence is essential to emphasize that we are excluding newborn pneumonia, which requires way different clinical management and decisions.

*For clarity, I would recommend restating your primary outcome to simply “required tertiary care referral.” Having the outcome as severe versus nonsevere, which is defined as requiring tertiary care referral or not, adds an extra step to the thought process and can be confusing.*

Response: We appreciate the recommendation to clarify the primary outcome. In the revised manuscript, we have already redefined the primary outcome to “Level of Care Severity,” scaled as severe or nonsevere, and defined it as the need for referral to a tertiary care unit for intensive care or respiratory support. This phrasing preserves the conceptual framework of care severity levels while directly specifying that the outcome reflects the requirement for tertiary care referral. We believe this approach balances clarity with the study’s structured outcome definitions. Additionally, this terminology is consistently used in the entire manuscript, including the Methods section, where we explicitly defined it in Table 1.

*One of my largest concerns in the initial manuscript was the timing of the variables. This is crucial when determining how useful the model could be. If the elements in Table 1 are measured on admission, or in the first 6‐12 hours of admission, the model could be very useful for patient care. If the elements were measured at any point during the hospitalization, it becomes much less useful. My worry is that the model was developed based on the elements’ presence at any point, meaning if the child had fever, cough, respiratory distress, and hypoxia at hour 48, then at hour 49 the model was able to predict the patient would need transfer, and the patient was transferred at hour 50—this is not helpful to clinicians. On the other hand, if the model predicts at hour 12 that a patient needs transfer, and then at hour 50 they transfer, that is potentially very helpful to clinicians. Without these details, I cannot recommend the publication of the manuscript.*

Response: Thank you for emphasizing the importance of timing in assessing the model’s clinical utility again. We have already clarified this point in the revised manuscript by specifying that all variables in Table 1 were recorded at the time of admission. As stated in Table 1, these variables were extracted from initial examination documents, not from any time from the hospitalization period, reflecting the presence/measurement of variables at admission. We believe that timings are adequately mentioned by the “at admission” or “at initial examination” phrases in Table 1. Only the primary outcome “Level of Care Severity” was extracted from medical records other than the initial time point, as it is necessary to encode whether or not a patient had advanced support during their hospital stay.

*It appears that the model was developed using the data from all 437 patients, and the results are presented following k-fold cross validation. It is standard practice to derive the model on a subset of the data (typically 70%‐80%) and then to test it on the remainder of the dataset to prevent overfitting and inflation of performance metrics. It does not appear that this was done. Despite having a small sample size, I believe this approach would lead to a more robust and generalizable model.*

Response: Thank you for highlighting this point regarding model validation. In the revised manuscript, we confirmed that a k-fold cross-validation approach was used on the entire dataset to address the limited sample size. To mitigate concerns of overfitting and enhance model generalizability, we initially split the data, setting aside 5% as a test set to prevent data leakage. The remaining data were then used in an 85%:15% split for training and validation. This approach was chosen to maximize the utility of our sample while ensuring a robust evaluation of model performance. Please refer to the subsections named Handling With the Imbalanced Dataset and Algorithms, where we have already addressed the reviewer’s concern, in the revised manuscript from the round 1 review.

##### Results

*The first paragraph contains many “nuts and bolts” details of model development, and these would be better positioned in the Methods section.*

Response: Again, we are deeply concerned that the reviewer may not be reading the revised manuscript from the round 1 review. These concerns have already been addressed. In the revised manuscript, the Results section begins with subsection named Study Population Characteristics.

*Both reviewers on the initial submission requested additional details describing the study population, and although the authors responded that they added these details, there are still none provided. It is essential to the understanding of the study results to know the characteristics of the patient population, and it should be a standard requirement for all clinical studies.*

Response: We have already agreed on this issue and carefully included a substantial revision with a Study Population Characteristics subsection and a detailed Table 2, reflecting the study population adequately. Please refer to these sections, and we are prepared to address any further concerns regarding the presentation of the study population if needed.

*The Shapley additive explanations value results presented in Figure 2 are valuable, but more details describing each measured factor are required. I recommend a table with each factor as rows and two columns comparing the population that did not require transfer to a tertiary care center to the population that did.*

Response: Again, this concern has already been addressed by Table 2, with a basic statistical comparison between two groups including test statistics with the significance level.

*An additional figure showing an area under the precision-recall curve for each model would also be interesting to the readers.*

Response: On the round 1 revision, we have already included a new figure in Multimedia Appendix 2, showing the PRC. This may have been spared from the reviewer’s eye.

##### Discussion

*The Discussion spends a decent amount of space discussing the COVID-19 pandemic. While this does have some bearing on the management of childhood pneumonia, I believe the space would be better spent discussing the actual implementation of this type of algorithm. How would a primary care clinician actually use this model in practice? How would it improve upon current clinical practice? Would it be easy or difficult to incorporate into routine workflows? This would be more interesting to the readers.*

Response: The revised manuscript has substantially been changed, reducing the amount of emphasis on the pandemic and carefully answering those questions that have been raised by the reviewer in the first round.

*I recommend adding what the next steps of this line of research would be. How would you seek to improve the model’s performance? More patient data? Additional variables?*

Response: We have provided recommendations along with our limitations. Please refer to our Limitation paragraph—specifically, just before the Conclusion paragraph.

*In the original submission, I recommended the authors provide a limitations section and also provided some examples. Although the authors response says they added this, there are still no limitations provided. Please provide this essential element to the Discussion.*

Response: This new comment provides evidence that the reviewer was not reading the revised manuscript from the first round, because we have one relatively long paragraph dedicated to the limitations of this study. The Limitation paragraph starts with “One significant limitation of this study…” We have double-checked the JMIRx submission system, and we confidently confirm that we have uploaded the revised manuscript correctly.

##### Conclusion

*I recommend commenting on what the next steps of this line of research would be in more specific terms.*

Response: We believe that our Conclusion reflects the primary findings of the study along with its clinical importance and applicability.

## Round 3 Review

### Reviewer E

#### General Comments

*The authors have conducted a single-center, retrospective study evaluating the derivation and performance of a machine learning model to predict the need for transfer to a higher level of care for childhood pneumonia. The authors were provided with a substantial amount of feedback on the original submission and have been responsive to feedback, which has resulted in a much improved manuscript. There remain several typographical and grammatical errors, which I would advise an English-grammar expert to review prior to publication, but from a scientific standpoint, I believe the manuscript is appropriate for publication.*

Response: We sincerely appreciate the reviewer’s recognition of the improvements made to the manuscript and their support for its scientific merit. We have carefully reviewed the manuscript for typographical and grammatical errors to ensure the highest standard of clarity and professionalism prior to publication. Thank you again for your valuable feedback that improved the quality of our work.

#### Specific Comments

##### Major Comments

1. *Details regarding the patient population have been provided in detail.*
2. *The study objectives have been clarified for readers.*
3. *The study methods are now much more reproducible.*

Response: These aspects were prioritized during the revision process, guided by the reviewers’ constructive feedback, which significantly enhanced our work. Their insightful comments not only improved this manuscript but also provided valuable lessons for our future works.

## Abbreviations

PRC: precision-recall curve
TRIPOD: Transparent Reporting of a Multivariable Prediction Model for individual Prognosis or Diagnosis
